# Deep forest model for diagnosing COVID-19 from routine blood tests

**DOI:** 10.1038/s41598-021-95957-w

**Published:** 2021-08-17

**Authors:** Maryam AlJame, Ayyub Imtiaz, Imtiaz Ahmad, Ameer Mohammed

**Affiliations:** 1 Department of Computer Engineering, Kuwait University, Kuwait City, Kuwait; 2 Saint Elizabeths Hospital, Washington, DC USA

**Keywords:** Infection, Health care

## Abstract

The Coronavirus Disease 2019 (COVID-19) global pandemic has threatened the lives of people worldwide and posed considerable challenges. Early and accurate screening of infected people is vital for combating the disease. To help with the limited quantity of swab tests, we propose a machine learning prediction model to accurately diagnose COVID-19 from clinical and/or routine laboratory data. The model exploits a new ensemble-based method called the deep forest (DF), where multiple classifiers in multiple layers are used to encourage diversity and improve performance. The cascade level employs the layer-by-layer processing and is constructed from three different classifiers: extra trees, XGBoost, and LightGBM. The prediction model was trained and evaluated on two publicly available datasets. Experimental results show that the proposed DF model has an accuracy of 99.5%, sensitivity of 95.28%, and specificity of 99.96%. These performance metrics are comparable to other well-established machine learning techniques, and hence DF model can serve as a fast screening tool for COVID-19 patients at places where testing is scarce.

## Introduction

The novel coronavirus disease 2019 (COVID-19) pandemic caused by severe acute respiratory syndrome coronavirus 2 (SARS-CoV-2) started affecting the globe around March 11th, 2020. Its spread across the world continues to pose serious challenges to human society with more than 126 million confirmed cases and around 2 million deaths^1^. The second wave of COVID-19 is further threatening the lives of large population of people. In order to combat the pandemic, it is of prime importance to quickly and accurately identify infected people and provide timely treatment by proper management and allocation of constrained healthcare resources. The current gold standard test for detection of COVID-19 is the Reverse-Transcription Polymerase Chain Reaction (RT-PCR)^2^ done on a swab of the nasopharynx/oropharynx. Despite being the current gold standard, the RT-PCR test has some shortcomings. These include being costly, user error prone, not easily accessible at all locations and a relatively long turnaround time^3^. It is thus imperative to develop alternative detection methods that are cheaper, simpler, faster and easier to be deployed to all locations while still maintaining a high accuracy for COVID-19. These alternative COVID-19 detection methods heavily rely on the epidemiological features, clinical characteristics, imaging findings (chest X-rays, CT scans) and standard laboratory tests (blood, urine)^4,5^.

To further improve and accelerate the COVID-19 early detection process, there is an emergent interest in exploring the potential of machine learning tools, especially for medical imaging (CT scans and chest X-rays)^6,7^ and routine laboratory and/or clinical data^8^. In recent studies^9–13^, routine laboratory and/or clinical data based early detection methods are being favoured as they are faster, easier to use, more accessible and less expensive alternatives when compared with medical imaging techniques. In this article, we address the use of machine learning techniques based on routine laboratory and/or clinical data for reliable early detection of COVID-19. Currently, deep neural networks (DNNs) are the most promising machine learning models, which are built upon multiple layers of parameterized differentiable non-linear neural network modules that can be trained by backpropagation^14^. However, DNNs suffer from some key deficiencies such as the tuning of too many hyper-parameters and poor interpretability. They also require a huge amount of data and expensive high-performance computing resources during the training process.

Recently, a new ensemble-based method called the deep forest (DF) was proposed by Zhou and Feng^15^ as an alternative to DNNs. It combines several ensemble-based methods with non-differentiable modules such as random forests^16^ and stacking. In contrast to DNNs, DF has fewer hyper-parameters, does not require backpropagation, is easy to train with low computational costs, and works well even for only small-scale training data. Since its inception, the DF algorithm^15^ has demonstrated an excellent performance in a wide range of applications in diverse fields such as diagnosing schizophrenia^17^, price prediction^18^, image retrieval^19^, drug interactions^20^, COVID-19 detection from CT images^21^, hyperspectral image classification^22^, human age estimation from face images^23^, short-term load forecasting of power systems^24^, and emotion recognition^25^ among others.

Seeing how successful the DF model can be in multiple fields, we decided to investigate the possibility of building a deep forest model based on non-differentiable modules such as random forests for initial screening of COVID-19. To date, DF based models have not been used for COVID-19 diagnosis from routine laboratory and/or clinical data. In this article, we tailored the DF based model for COVID-19 diagnosis problem with the aim to achieve better performance as compared with DNNs. The proposed model exploits the advantages of the DF as an ensemble approach where multiple classifiers are used to encourage diversity and improve performance. The cascade level in the proposed DF based model employs the layer-by-layer processing. It is constructed from three different classifiers: extra trees, XGBoost, and LightGBM. The experimental results show that the proposed DF based model achieved considerable improvement in performance metrics for diagnosing COVID-19 as compared with recently proposed DNNs model^13^ for a publicly available dataset from Albert Einstein Hospital in Brazil^26^. Further comparisons are accomplished against recently proposed techniques^9,10,27^ for the IRCCS Ospedale San Raffaeleis dataset^10^.

The remainder of the paper is structured as follows: “Related work” section reviews the related work in the field. The proposed deep forest based model is described in “Design methodology” section  and experimental results are discussed in “Performance evaluation and discussions” section. Finally, concluding remarks and future work are made in “Conclusions” section.

## Related work

A review of the machine learning models for diagnosing COVID-19 using routine laboratory and/or clinical data can be found in article^12^. The most popular prediction models proposed were based on random forest (RF)^16^, logistic regression (LR)^28^, support vector machine (SVM)^29^ and neural networks (ANN)^30^. In this section, we describe the recent machine learning models reported for early detection of COVID-19 or identifying the severity level of confirmed COVID-19 patients based on laboratory and/or clinical data.

Cabitza et al.^9^ extended their earlier work reported in^31^ for COVID-19 detection from routine blood samples. The authors considered five machine learning models including RF, naive bayes (NB), LR, SVM, and k-nearest neighbors (KNN). A dataset of 1,624 routine blood samples was obtained from patients (52% COVID-19 positive) admitted to San Raphael Hospital (OSR), Milan, Italy. The models resulted in an accuracy in the range of 74–88%, a sensitivity in the range of 70–89%, a specificity in the range of 79–92% and an Area Under the Curve (AUC) in the range of 74–90%.

Abdulaal et al.^11^ designed and compared an artificial neural network (ANN) and COX regressions models based on clinical data to predict the death for COVID-19 patients. The clinical dataset is collected from 398 patients in London Teaching Hospital. The Cox regression model achieved an average accuracy of 83.8%, a sensitivity of 50%, a specificity of 96.6% and an AUC of 86.9%, whereas ANN model accuracy was 90%, a sensitivity of 64.7%, a specificity of 96.8% and an AUC of 92.6%.

An ensemble learning model for COVID-19 diagnosis from routine blood tests was proposed in^12^. The ensemble model used three classifiers extra trees, RF and LR at the first level, and an extreme gradient boosting (XGBoost) classifier at the second level to combine the predictions from the first level classifier. The model was trained and evaluated by using a dataset from Albert Einstein Hospital in Brazil^26^.

In a recent study^13^, the authors were the first ones to report the use of deep learning models to diagnose COVID-19 from routine blood tests. The authors selected six different deep learning models including ANN, convolutional neural networks (CNNs), long-short term memory (LSTM), recurrent neural networks (RNNs), CNNLSTM, and CNNRNN for evaluation. Models were trained and tested with 18 blood features from 600 patients seen at the Albert Einstein Hospital in Brazil^26^. The best performing algorithm was the CNNLSTM hybrid model with train-test split approach, which resulted in an accuracy of 92.3%, a precision of 92.35%, an AUC of 90%, a F1-score of 93% and a recall of 93.68%.

Aktar et al.^32^ addressed the problem of identifying the severity level of confirmed COVID-19 patients for their appropriate ward selection (intensive care units (ICUs) or normal ward) based on blood samples. The authors considered eight machine learning models including decision tree (DT), RF, gradient boosting machine (GBM), XGBoost, SVM, light gradient boosting machine (LGBM), KNN, and ANN. The models resulted in an accuracy and a precision of above 90% for disease severity and mortality predictions.

Yao et al.^33^ investigated the detection of severity level of COVID-19 patients by using the clinical information and the blood/urine test data. The authors utilized five machine learning models including LR, RF, Adaboost, SVM, and KNN. The dataset consisted of 137 clinically confirmed cases of COVID-19 patients from Tongji Hospital, China. Among the models, SVM was the winner which achieved an overall accuracy of 81.48%. Henzel et al.^34^ used LR and XGBoost predictive models for the early screening of COVID-19 patients. Dataset consisted of 3114 patient’s records collected at a hospital in Poland was used to train and test the models.

In contrast to other studies, Razavian et al.^35^ developed a model with primary focus to identify COVID-19 patients with favourable outcomes within three days of a prediction. The aim of the study was to discharge low risk patients to free up limited beds for incoming patients. The authors trained four classifiers: LR, RF, LGBM and ensemble of these three models based on a simple averaging of the model probabilities. The models achieved a high average precision of 88.6%.

Hallman et al.^36^ utilized XGBoost prediction model to determine which level of care a COVID-19 patient requires such as self-quarantine, admitted to the hospital or sent to ICU. The model was trained with 70% data and tested with 30% data from Albert Einstein Hospital in Brazil^26^.

Goodman-Meza et al.^37^ developed a prediction model for screening the COVID-19 patients based on demographic and laboratory features. The authors considered seven classifiers including RF, LR, SVM, multilayer perceptron (neural network), stochastic gradient descent, XGBoost, and ADABoost. Furthermore, an ensemble model was created based on the seven models, where the final classification was decided by using the majority vote of the classifiers. The dataset was collected from the UCLA Health System in Los Angeles, California. The ensemble model achieved a sensitivity of 93%, a specificity of 64% and an AUC of 91%.

Chao et al.^38^ used RF model to predict the need for COVID-19 patient for ICU admission based on both imaging (lung) and non-imaging features from demographic data, vital signs, and laboratory findings. The proposed model was trained and evaluated on datasets of 295 COVID-19 patients collected from three different hospitals (one in the United States, one in Iran, and another in Italy). The model achieved a sensitivity of 96.1% and an AUC of 88.4%.

Wang et al.^39^ proposed a model to predict three clinical outcomes: deceased, ventilated, or admitted to ICU for COVID-19 positive patients at NYU Langone Health (NYULH). The authors considered two prediction models: LR with feature selection using Least Absolute Shrinkage and Selection Operator (LASSO) and XGBoost. Models were trained with a dataset of 3740 patients and XGBoost model excelled in performance as compared with LR.

Vaid et al.^40^ utilized XGBoost classifier model to predict in-hospital mortality and critical events at time windows of 3, 5, 7, and 10 days from admission. The model was trained and validated by using the electronic health records (EHRs) of COVID-19 positive patients admitted to Mount Sinai Health System in New York City. The XGBoost model achieved good performance for mortality as well as for critical event prediction.

Zhu et al.^41^ developed a 6-layer deep neural network to identify 5 top variables among 56 clinical variables at admission to predict the likelihood of mortality in COVID-19 patients. Data of 181 patients was collected from a major hospital in Wuhan, China to train and test the model. Prathamesh et al.^42^ developed a RF model for the prediction of near-term (20–48 h) mortality based on time-series inpatient data from electronic health records. The dataset of 567 patients was collected from hospital in New York.

The authors in^43^ proposed a model to predict the survival of COVID-19 positive patients in the region of Madrid, Spain. The authors utilized LR, DT, RF, BN and clustering machine learning models for classification. The LR model achieved the best predicting performance in testing. Wu et al.^44^ developed a prediction model to assess the severity risk of COVID-19 positive patients based on clinical features. The dataset of 725 patients was collected from eight different centers in China, Italy and Belgium. The authors trained and validated four models of LR, each model with a different subset of features from the dataset.

Yue et al.^45^ reported a mortality risk model for COVID-19 based on clinical data in EHRs from hospitals in China. The authors proposed an ensemble model based on four known classifiers including LR, SVM, GBM, and ANN. The model achieved good performance on both internal and external validation. The authors in^46^ proposed mortality prediction model among confirmed COVID-19 patients in South Korea. Five machine learning algorithms (LR, SVM, KNN, RF and GBM) were used for prediction. The LR algorithm achieved the best performance.

Connor et al.^47^ proposed models to predict hospitalization within 4 weeks of an outpatient COVID-19 test, ICU admission, mechanical ventilation and inpatient mortality by leveraging EHR data from the Duke University Health. For each type of models, three classifiers were considered including logistic regression, XGBoost and LGBM. Based on the validation, LGBM performed the best.

Casiraghi et al.^48^ built an explainable COVID-19 risk prediction model based on clinical, laboratory and radiological data. First the features were selected based on their importance and then a RF classifier was trained with the chosen features. Kenneth et al.^49^ developed a model to predict the risk of developing severe or fatal infections based on the UK Biobank. XGboost machine learning model was trained with 93 clinical variables and its predictive performance was assessed by cross-validation.

Xu et al.^50^ developed a multi-class classification model to predict non-severe COVID-19, severe COVID-19, non-COVID viral infection, and healthy classes from clinical, lab testing, and CT scan features. A deep CNN was used to extract features from CT scan. Then, features from three different modalities (clinical, lab testing and CT scan) were fused together to train three machine learning models (KNN, RF, and SVM) to differentiate four classes at once. All three models achieved high accuracy (95.4–97.7%) to differentiate the overall four classes.

Souza et al.^51^ proposed a model to study the disease progression in positive COVID-19 patients based on demographic and clinical data along with comorbidities in Brazil. The authors trained and evaluated seven machine learning models including LR, Linear Discriminant Analysis (LDA), NB, KNN, DT, XGBoost and SVM to predict the disease outcome. Chen et al.^52^ investigated COVID-19 severity by using RF based on 26 comorbidity/symptom features and 26 blood features from patients in Wuhan, China. The authors identified the top five features from each modality to train and validate the model.

Bezzan et al.^53^ selected XGBoost model using Bayesian Optimization among several machine learning models to predict whether COVID-19 patients are going to require special care (hospitalisation in regular or special-care units). The model was trained and evaluated based on lab exam data from patients in different hospitals in Brazil.

Subudhi et al.^54^ compared the performance of 18 machine learning algorithms for predicting ICU admission and mortality among COVID-19 patients. The evaluated 18 machine learning algorithms belong to 9 broad categories, namely ensemble, gaussian process, linear, naïve bayes, nearest neighbour, support vector machine, tree based, discriminant analysis and neural network models. The dataset was obtained from 10,826 COVID-19 positive patients in the multihospital database (Massachusetts General Brigham Healthcare database). The ensemble-based models achieved the best performance among all the models.

Fakhartousi and Davies^27^ reported a framework to select the best set of features from all the features obtained from routine blood tests with the aim to improve the COVID-19 diagnosis. For their study, the data samples of 279 patients from IRCCS Ospedale San Raffaele was collected, which included 177 positive samples and 102 negative samples. The authors employed 6 prediction models including KNN, LR, DT, RF, SVM, and NB. The authors considered different features selection methods to enhance the model prediction accuracy. Experimental results demonstrated that the combination of proper feature selection and prediction model played a key role for reliable and accurate detection of the coronavirus.

A review of machine learning prediction models for COVID-19 diagnosis is given in Table 1. The six most popular classifiers employed in literature are the RF, LR, SVM, XGBoost, KNN and DNNs. As one can observe from Table 1, that DF has not been studied for COVID-19 diagnosis. The aim of this work is to study and compare the performance of DF with the existing techniques for COVID-19 detection. The structure of the deep forest enhances COVID-19 prediction as it is built based on accurate and diverse set of non-differentiable classifiers such as RF, and XGBoost. This work exploits the strength of DF to build a better COVID-19 prediction model on different datasets.

**Table 1:** Summary of machine learning prediction models for COVID-19 diagnosis.

Model	References
COX Regression	^11^
Linear Discriminant Analysis (LDA)	^50,53^
AdaBoost	^32,36^
Light Gradient Boosting Machine (LGBM)	^31,34,46^
Gradient Boosting Machine (GBM)	^31,44,45^
Naïve Bayes (NB)	^9,10,27,42,50,53^
Decision Trees (DTs)	^12,27,31,42,50,53^
Deep Neural Networks (DNNs) [ANN, CNN, LSTM, RNN]	^11,13,31,36,40,44,49,53^
K-Nearest Neighbour (KNN)	^9,10,27,31,32,45,49,50,53^
Support Vector Machine (SVM)	^9,10,27,31,32,36,44,45,49,50,53^
Extreme Gradient Boosting (XGBoost)	^12,31,33,35,36,38,39,46,48,50,52^
Logistic Regression (LR)	^9,10,12,27,32–34,36,38,42–46,50,53^
Random Forest (RF)	^9,10,12,27,31,32,34,36,37,41,42,45,47,49,51,53^

## Design methodology

### Dataset description

Two different datasets were used for this study, the first is publicly available by Kaggle^26^. This dataset has 5644 patient records which were collected from the 28th of March 2020 to 3rd of April 2020 at the Albert Einstein Israelita Hospital located in São Paulo, Brazil. The Albert Einstein dataset is informative as it contains several clinical tests data such as blood, urine, SARS-CoV-2, and rt-PCR test. The clinical data were standardized to have a mean of zero and a unit standard deviation. In this dataset 559 patients received a positive diagnosis while 5085 were negative cases. The SARS-Cov2 attribute indicates COVID-19 diagnosis, the proposed model converted the SARS-Cov2 attribute from string to integer where it is equal to zero in the absence of COVID-19 infection, and it is equal to one for positive cases. The second dataset is publicly available by the IRCCS Ospedale San Raffaele^10^. This dataset has 279 patients who were admitted to San Raffaele Hospital, Milan, Italy, from the end of February 2020 to mid of March 2020. The patient information in the San Raffaele dataset includes age, gender, routine blood tests values, rt-PCR test, to name a few. Among 279 patients, 177 patients were diagnosed as positive cases, the Swab variable gives COVID-19 diagnosis.

### DF-COVID-19 model general pipeline

**Figure 1:**
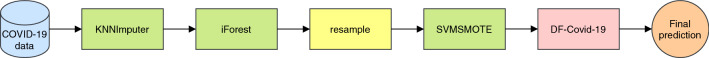
A general pipeline of DF-COVID-19 model.

The general pipeline of DF-COVID-19 model is illustrated in Fig. 1. The green rectangles represent data preparation steps. In this study, there are three steps to prepare data. The first step is handling missing values where KNNImputer is utilized from sklearn.impute Python library. For imputation, KNNImputer used the k-nearest neighbors algorithm. The number of nearest neighbors is determined by n_neighbors parameter which was set to 11 in this work. The second step is removing outliers with isolation forest (iForest)^55^ from sklearn.ensemble Python library^56^. Removing outliers improves the classification model performance because there are quantity and quality differences between outliers and normal records. The initial step iForest follows to remove outliers is creating an ensemble of isolation trees (iTrees). Then, detecting outliers is done by computing the average path lengths for each instance on the iTrees, outlier has a short average path length. The proposed model tunes two parameters of iForest: n_estimators and contamination. The number of estimators has been set to 150. The contamination parameter controls the outliers ratio in the dataset. The contamination parameter has been set to 2%. After that, from sklearn.utils Python library resample is employed to create small multiple subsets with replacement from the entire dataset. This resampling step enhances data variety and randomness. Thereafter, the whole dataset is randomly split into the training set (80%) and the test set (20%). The third step in data preparation is balancing the training data. This is an important step to avoid the bias of classifying toward the majority class. To achieve class balance, the dataset has been processed with SVMSMOTE^57^. The SVMSMOTE is an over-sampling technique using the SVM algorithm to generate new synthetic samples of the minority class. The proposed model applied SVMSMOTE from imblearn.over_sampling Python library with 11 neighbors. After all the aforementioned steps, the data is processed into the DF-COVID-19 model. Finally, the final output is the predicted value of COVID-19 diagnosis which is either positive or negative.

### The DF-COVID-19 model

The Deep Forest introduced in^15^ has proved its robustness in classification problems. This inspired us to develop DF-COVID-19, a prediction model to predict COVID-19 diagnosis from routine blood tests. The proposed model code is based on the code of the Deep Forest open source^58^.

**Figure 2:**
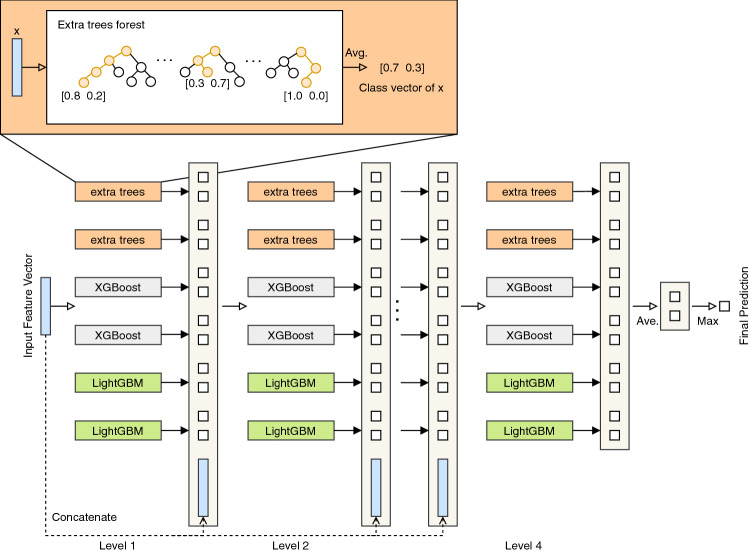
Illustration of the cascade forest structure of DF-COVID-19 model.

Figure 2 illustrates the structure of the cascade level in the proposed model. The structure is composed of different types of forests that encourage the diversity of classifiers. In fact, diversity helps in increasing the accuracy of an ensemble model. The DF-COVID-19 cascade level is constructed using six forests: two extra trees, two XGBoost, and two LightGBM. The number of trees in each forest is 250, and for each tree the maximum depth was set to 7. One of the main advantages of a deep forest model is that it has few hyper-parameters to tune. For instance, the parameter max_layers determines the maximum number of cascade layers. By setting this parameter, the structure of any deep forest based model is designed to check if adding a new cascade level will improve the performance or not, then this checking specifies the automatic termination of the cascade levels expansion progress. As shown in Fig. 2, the number of cascade levels in the proposed model is four. Figure 2 depicts that each forest in the cascade outputs a two dimensional class vector. In our case, the two dimensional class vector has two values: positive and negative COVID-19 diagnosis. The model processes the dimensional vector layer-by-layer until the last layer to predict the final prediction. Thus, each layer in the cascade receives its input by its preceding layer, and outputs its results to the next layer. In detail, every single forest in the model will produce a 2-dimensional class vector. As a result, the generated class vectors of all forests in the DF-COVID-19 are equal to 12 where each forest generates one 2D vectors so 6 vectors in total, as illustrated in Fig. 2. After that, the generated class vectors are concatenated with the original input feature vector to be processed to the next level. To lower the risk of overfitting during the generation of every class vector, the model uses k-fold cross-validation. Subsequently when the last level in the cascade finishes its processing, the information produced by every forest in the previous layer will be averaged resulting in generating the final class vector. The final prediction is the maximum value in the final class vector.

The upper left corner in Fig. 2 illustrates an example of generating class vector for the extra trees forest. The leaf node has the concerned instance, for each forest an estimate vector of class distribution is created by calculating the percentage of different classes at the leaf node. Then, the final class vector in the last layer is computed by averaging all trees in the same forest, as shown in Fig. 2.

### Feature importance

**Figure 3:**
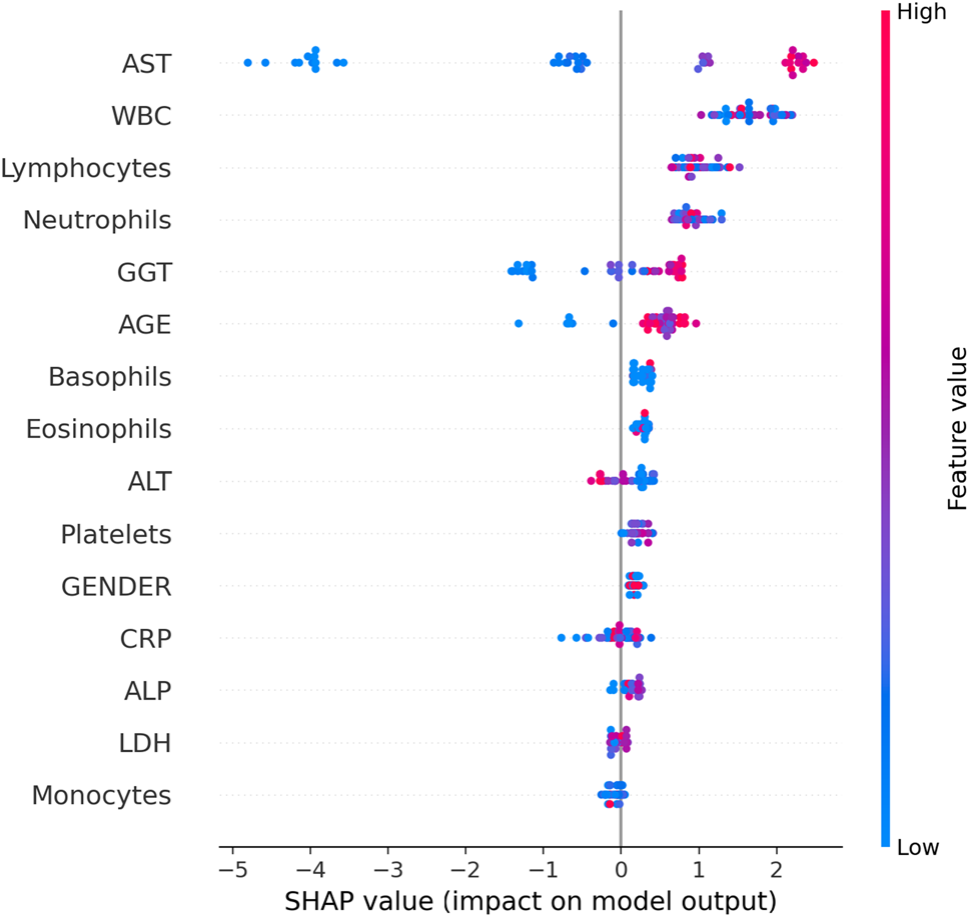
SHAP summary plot for a LightGBM classifier in the DF-COVID-19 model.

The prediction of a classification model is processed based on different factors. An important step in the classification process is selecting the appropriate features. Feature importance measures how a feature affects a prediction with a magnitude and a direction either positive or negative. There are several approaches used to determine the feature importance. In this study, the SHapley Additive exPlanations (SHAP)^59^ is utilized to assess the importance of a feature in predicting COVID-19. SHAP is a technique based on a game theory that produces an interpretable model by using Shapley values. Since DF-COVID-19 is constructed of deep forest which is an ensemble of tree-based models, the TreeExplainer^59^ is applied to compute the SHAP values of the proposed model. Figure 3 depicts the SHAP summary plot for one of the LightGBM classifiers in the cascade level of the DF-COVID-19 model. In this example, the selected features are set to be the same as the study^60^. The SHAP summary plot reveals the impacts of input features on COVID-19 positive cases prediction. The y-axis lists the input features in a descending order according to their importance. Each point on the plot represents a Shapley value, the color of the point ranges from blue (low) to red (high). The density of the points indicates the distribution in the dataset. Figure 3 shows that aspartate aminotransferase (AST) is the most important factor. The next critical features are white blood cells counts (WBC), lymphocyte count (Lymphocytes), neutrophil count (Neutrophils), gamma glutamyl transpeptidase (GGT), and age. The SHAP summary plot proves that low values of WBC, lymphocyte and neutrophil tend to increase the probability of positive diagnosis. While high values of GGT and age increase the possibility of COVID-19. Figure 3 demonstrates that a decrease in basophils count (Basophils), eosinophil count (Eosinophils) and alanine aminotransferase (ALT) leads to a decrease in the possibility of COVID-19 positive cases. As shown in Fig. 3 the least significant features are: c-reactive protein (CRP), alkaline phosphatase (ALP), lactate dehydrogenase (LDH), and monocytes count (Monocytes).

## Performance evaluation and discussions

### Performance metrics

Evaluating the model performance is one of the important steps. This study adopted five metrics to assess the performance of the proposed model: accuracy, AUC, sensitivity and specificity. Confusion matrix values are used to calculate the aforementioned performance metrics. The confusion matrix values include: true positive (TP), false positive (FP), true negative (TN), and false negative (FN). TP and TN are when the actual class is correctly predicted, while FP and FN are when the actual class is incorrectly predicted. The area under the ROC Curve is known as AUC which illustrates the relation between TP (y-axis) and FP (x-axis). High score of AUC indicates that the model has high quality in differentiating its classes. The proportion of correctly predicted cases (TP and TN) over the whole dataset predictions is measured by accuracy. This measurement is mathematically described by the following Eq. (1):

1 $$\begin{aligned} Accuracy = \dfrac{TP+TN}{TP+TN+FP+FN}*100 \end{aligned}$$

Specificity is defined as the proportion of true negative. The higher value of specificity the model has the better the model performs. The formula of specificity is given by Eq. (2):

2 $$\begin{aligned} Specificity = \dfrac{TN}{TN+FP} \end{aligned}$$

Sensitivity is the proportion of true positive. For instance, the proportion of COVID-19 infection that the model correctly predicted them as positive cases. The following Eq. (3) computes the sensitivity:

3 $$\begin{aligned} Sensitivity = \dfrac{TP}{TP+FN} \end{aligned}$$

Precision is an evaluation metric known as Positive Predictive Value (PPV). The following formula measures the precision:

4 $$\begin{aligned} Precision = \dfrac{TP}{TP+FP} \end{aligned}$$

F-Measure or F1-score analyses a model based on the precision and recall in one metric by computing their harmonic means using the following equation:

5 $$\begin{aligned} F1-score = \dfrac{2TP}{2TP+FP+FN} \end{aligned}$$

### Comparison with other classification models

In this section, the DF-COVID-19 model is evaluated by comparing it with other state-of-the-art approaches^13,27,60–64^. For comparison fairness, both DF-COVID-19 and previous studies used the same dataset. In addition, for the proposed model the selected features are set exactly as the study against which the results were compared. The results of the previous approaches used for comparison are the ones reported in their study. DF-COVID-19 results are the average of 100 repetitions and the 95% confidence interval are calculated by bootstrapping the dataset. This section conducts four experiments, where the best result is highlighted as bold in the tables.

The first experiment is a comparison with the study in^13^. This study predicts COVID-19 diagnosis by training several deep learning approaches including: artificial neural network (ANN), convolutional neural networks (CNNs), long-short term memory (LSTM), and recurrent neural networks (RNNs). In addition, two hybrid deep learning models were implemented: CNNLSTM and CNNRNN. The training or evaluation were performed with two different approaches: 10 fold cross-validation and 80-20 train-test split. The Albert Einstein dataset^26^ is used in this experiment. Table 2 reports the comparison results between DF-COVID-19 model and the deep learning models in^13^. It can be observed that DF-COVID-19 achieved the best results in the terms of accuracy and AUC. It is worth mentioning that the six different deep learning models in^13^ have fluctuated values of AUC. For example, the highest value is 90% recorded by CNNLSTM with train-test split approach while the lowest value is 52.45% achieved by RNN with 10 fold cross-validation approach. Those lower values of AUC indicate that the deep learning models have limitations in distinguishing between positive and negative cases of COVID-19. Further, the best precision of 92.35% is obtained by CNNLSTM using train-test split approach. On the other hand, the DF-COVID-19 model recorded a lower precision of 85.66%. In terms of sensitivity and F1-score, the LSTM and CNNLSTM classifiers are mostly better than the DF-COVID-19. However, DF-COVID-19 proved its effectiveness in terms of accuracy, AUC, and precision.

**Table 2:** The comparison results between DF-COVID-19 and other deep learning models.

Model	Accuracy	AUC	Precision	Sensitivity	F1-score
ANN in^13^ train-test split	86.90%	85.0%	87.13%	87.13%	87.13%
10 fold cross-validation	86.0%	56.15%	88.55%	95.78%	91.34%
CNN in^13^ train-test split	87.35%	80.0%	88.47%	88.67%	88.56%
10 fold cross-validation	88.0%	61.49%	89.48%	92.48%	90.38%
CNNLSTM in^13^ train-test split	92.30%	90.0%	**92.35%**	93.68%	**93.0%**
10 fold cross-validation	84.16%	58.89%	89.26%	92.14%	90.01%
CNNRNN in^13^ train-test split	86.24%	69.0%	87.55%	87.55%	87.55%
10 fold cross-validation	85.66%	64.08%	89.77%	94.23%	91.20%
LSTM in^13^ train-test split	90.34%	83.0%	89.97%	89.98%	89.97%
10 fold cross-validation	86.66%	62.50%	86.75%	**99.42%**	91.89%
RNN in^13^ train-test split	84.0%	83.0%	84.28%	84.27%	84.27%
10 fold cross-validation	84.16%	52.45%	87.83%	96.04%	90.61%
**DF-COVID-19** with^13^ features	**93.98%** [95%CI 88.29–100]	**94.91%** [95%CI 84.1–99.8]	85.66% [95%CI 56.3–100]	66.3% [95%CI 35.4–92.3]	73.33% [95%CI 48.5–92.0]

For the second experiment, Table 3 shows the comparison of the proposed method with the different combinations of selected features and models in^27^. Different subsets of features are employed in^27^ with the aim of selecting the best set of features that enhances the prediction of COVID-19 infection. Those subsets of features are: weighted by information gain ratio (Wei_IGR), weighted by correlation (Wei_Cor), forward, backward, and optimize. The forward features obtained the best accuracy and sensitivity with RF and SVM, respectively. The best specificity is achieved by NV with Wei_IGR features. The IRCCS Ospedale San Raffaele^10^ dataset is used in this experiment. Among the different subsets of features, the best results of DF-COVID-19 are accomplished by applying Wei_IGR subset. Results in Table 3 demonstrate the improvement of DF-COVID-19 in accuracy and specificity. However, DF-COVID-19 recorded much lower sensitivity.

**Table 3:** The comparison results between DF-COVID-19 and other classifiers.

Model	Accuracy	Sensitivity	Specificity
DF-COVID-19 with^27^ Wei_IGR selected features	**88.16%** [95%CI 78.41–100]	91.27% [95%CI 75.1–100]	**81.98%** [95%CI 57.3–100]
Classifiers in^27^	80.3% RF forward selected features	**98.28%** SVM forward selected features	79.17% NV Wei_IGR selected features

**Figure 4:**
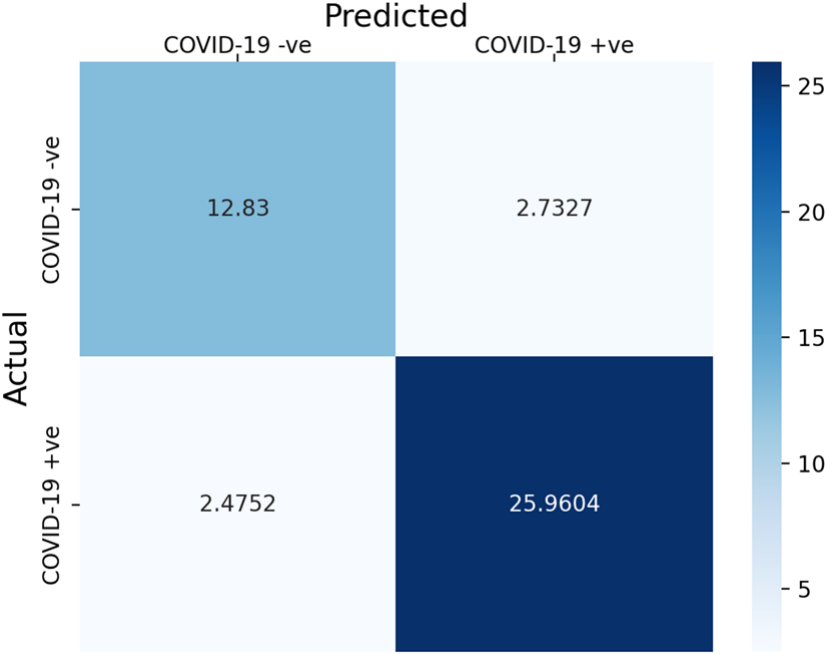
Average confusion matrix of DF-COVID-19 with Wei_IGR selected features.

Figure 4 shows the average confusion matrix obtained from 100 runs of DF-COVID-19 with the selected features: Wei_IGR^27^. On average DF-COVID-19 predicted correctly 12.83 of COVID-19 negative cases and 25.9604 of COVID-19 positive cases. Those values are the true negative and true positive. On the contrary, the false values are low as illustrated in Fig. 4. The false negatives average is 2.4752, and the false positives average is 2.7327. Thus, the confusion matrix values provide evidence for the robustness of the proposed model.

**Figure 5:**
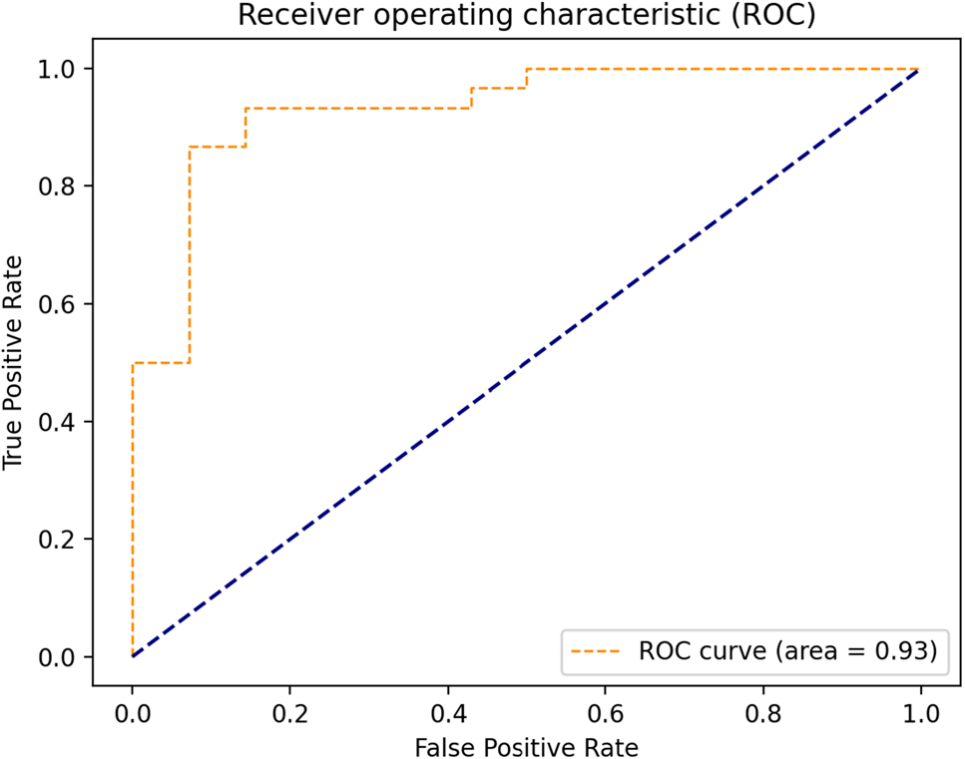
The ROC curve of DF-COVID-19 for the test set.

In the third experiment, the performance of DF-COVID-19 is compared with the strategy in the study^60^. The dataset used in this experiment is taken from San Raffaele Hospital in Milan^10^. The Feature Correlated Naïve Bayes (FCNB) is introduced in^60^ to diagnosis COVID-19 in an accurate and a fast way. FCNB has four phases: Feature Selection Phase (FSP), Feature Clustering Phase (FCP), Master Feature Weighting Phase (MFWP), and Feature Correlated Naïve Bayes Phase (FCNBP). FCNB took the advantages of the Naïve Bayes algorithm with introducing several modifications to it such as the correlation between features. The Feature Selection Phase (FSP) selects the most important features based on a wrapper method of genetic algorithm. The study^60^ reported the results of two scenarios which are: the FSGA and the FCNB strategy. Table 4 shows the comparison between DF-COVID-19, FSGA and FCNB. The best accuracy is obtained by FCNB which is 99.0%, FSGA has almost the same accuracy (98.0%) while DF-COVID-19 recorded on average a lower accuracy of 87.80%. However, DF-COVID-19 outperforms FSGA and FCNB in terms of precision, sensitivity, and F1-score. DF-COVID-19 achieved a similar precision with no significant difference when comparing it with FSGA. For sensitivity, DF-COVID-19 obtained a high sensitivity of 91.3%, contrastingly FSGA and FCNB got much lower sensitivity of 84.0% and 79.0%, respectively. In regard to F1-score, DF-COVID-19 achieved a significantly higher F1-score (81.72%) than FSGA (78.0%) and FCNB (76.0%). Moreover, the Receiver Operating Characteristic (ROC) of DF-COVID-19 is illustrated in Fig. 5. As shown in Fig. 5, DF-COVID-19 achieved a considerably high value of ROC equals to 0.93 which proves the model robustness in differentiating between COVID-19 cases. All results in this experiment confirm the effectiveness of DF-COVID-19 in selecting discriminative features that enhance model performance. One possible reason is the deep forest model on which DF-COVID-19 is based.

**Table 4:** The comparison results between DF-COVID-19, FSGA and FCNB.

Model	Accuracy	Precision	Sensitivity	F1-score
DF-COVID-19 with^60^ features	87.80% [95%CI 76.14–100]	**89.56%** [95%CI 75.4–100]	**91.3%** [95%CI 76.9–100]	**81.72%** [95%CI 56.5–100]
FSGA^60^	98.0%	89.0%	84.0%	78.0%
FCNB^60^	**99.0%**	84.0%	79.0%	76.0%

The last experiment in this section demonstrates the effectiveness of DF-COVID-19 against various studies^61–64^. Table 5 lists the comparison between DF-COVID-19 and the other studies which used the Albert Einstein dataset^26^. DF-COVID-19 dramatically outperformed ER-CoV^61^, ANN with SMOTE^62^, Bayes Net^63^, and SVM^64^ in terms of accuracy, AUC, and specificity. However, DF-COVID-19 and Bayes Net^63^ achieved very similar sensitivity.

**Table 5:** The comparison results between DF-COVID-19 and other studies.

Model	Accuracy	AUC	Sensitivity	Specificity
DF-COVID-19 with^61^ features	99.50% [95%CI 99.09–100]	99.91% [95%CI 99.7–100]	95.2% [95%CI 90.9–98.8]	99.96% [95%CI 99.7–100]
ER-CoV^61^	–	86.78% [95%CI 85.65–87.9]	70.25% [95%CI 66.57– 73.12]	85.98% [95%CI 84.94–86.84]
DF-COVID-19 with^62^ features	99.48% [95%CI 98.98–100]	99.91% [95%CI 99.3–100]	95.23% [95%CI 90.1–99.0]	99.94% [95%CI 99.7–100]
ANN with SMOTE^62^	87%	80 ± 0.09	43%	91%
DF-COVID-19 with^63^ features	99.49% [95%CI 98.98–100]	99.95% [95%CI 99.8–100]	95.28% [95%CI 89.8–99.0]	99.95% [95%CI 99.6–100]
Bayes Net^63^	95.159 ± 0.693	–	96.8 ± 0.007	93.6 ± 0.011
DF-COVID-19 with^64^ features	99.45% [95%CI 98.87–100]	99.91% [95%CI 99.6–100]	95.06% [95%CI 89.9–98.8]	99.92% [95%CI 99.6–100]
SVM^64^	–	85%	86%	85%

### Discussion

The recent outbreak of COVID-19 impacted human society with serious challenges. An early detection of COVID-19 patients yield to a significance prevention of the disease spreading. This study introduced DF-COVID-19 a coronavirus classification model that helps in COVID-19 diagnosis. There are several advantages in the proposed model one of them is a low computational cost when training data. In fact, deep forest based model requires a lower training stage complexity than a deep learning approach. In addition, the input of DF-COVID-19 is routine blood tests which are reasonable and prompt. Hence, the proposed model is considered affordable for low-income countries. Another advantage is DF-COVID-19 could be utilized as a reliable COVID-19 diagnosis model because the experimental results imply that the features selection of a deep forest based model enhances the model ability to diagnose COVID-19.

Although DF-COVID-19 achieves a competitive performance in COVID-19 classification, it has several limitations. First, DF-COVID-19 model is only able to predict COVID-19 diagnosis: whether it is positive or negative. Actually, more COVID-19 classification tasks are needed to be classified such as COVID-19 severeness detection, predict the need for intensive care unit (ICU) admission, and COVID-19 fatalities prediction. The second limitation is the size of the dataset used which is relatively small, this may limit the performance of the model. To overcome this limitation, a bigger dataset with more number of patients is recommended to be used to train and test the proposed model. Another limitation is the lack of dataset diversity, even though two different datasets are used in the current study, however the model generalizability have to be increased by utilizing more datasets. In addition, the model needs to be explored further including checking its ability to work on extremely imbalance dataset.

## Conclusions

The COVID-19 infection rate is still growing rapidly and continues to pose a dangerous threat to global health. Early catching of COVID-19 patients is crucial to combat the disease. However, PCR-based testing has bottlenecks in capacity and in scalability. In this article, a deep ensemble framework based on DF was exploited using routine laboratory and/or clinical data for fast screening of COVID-19 patients in hospital settings where PCR testing is scarce. In the proposed DF-based model, extra trees, XGBoost, and LightGBM were selected as the basic forests in each layer to improve the diversity to enhance performance. The training process is quick as it only has a few hyper-parameters to tune and does not require backpropagation and gradient adjusted, as compared with deep neural networks models. Experimental results showed that this DF-model has superior performance on COVID-19 diagnosis in comparison with existing state-of-the-art machine learning methods using information from two publicly available datasets. While computational methods come with their own limitations, application of this DF-based model would allow clinicians to have a quantifiable pre-test probability of COVID-19 cases, which can facilitate management, prognostication, and resource allocation. For the future work, we plan to extend the DF-based model to predict progression to severe COVID-19, respiratory insufficiency, need for ICU-level care, etc. in order to instruct more prompt management based on the availability of the dataset. In addition, the structure of the proposed model will be improved by including multi-grained scanning.
